# Analysis of Differentially Expressed Genes and Molecular Pathways in Familial Hypercholesterolemia Involved in Atherosclerosis: A Systematic and Bioinformatics Approach

**DOI:** 10.3389/fgene.2020.00734

**Published:** 2020-07-15

**Authors:** S. Udhaya Kumar, D. Thirumal Kumar, R. Bithia, Srivarshini Sankar, R. Magesh, Mariem Sidenna, C. George Priya Doss, Hatem Zayed

**Affiliations:** ^1^School of Biosciences and Technology, Vellore Institute of Technology, Vellore, India; ^2^Department of Biotechnology, College of Biomedical Sciences Technology and Research, Sri Ramachandra Institute of Higher Education and Research (DU), Chennai, India; ^3^Department of Biomedical Sciences, College of Health and Sciences, Qatar University, QU Health, Doha, Qatar

**Keywords:** familial hypercholesterolemia, atherosclerosis, coronary artery disease, functional enrichment analysis, expression profiling data, gene expression arrays

## Abstract

**Background and Aims:** Familial hypercholesterolemia (FH) is one of the major risk factor for the progression of atherosclerosis and coronary artery disease. This study focused on identifying the dysregulated molecular pathways and core genes that are differentially regulated in FH and to identify the possible genetic factors and potential underlying mechanisms that increase the risk to atherosclerosis in patients with FH.

**Methods:** The Affymetrix microarray dataset (GSE13985) from the GEO database and the GEO2R statistical tool were used to identify the differentially expressed genes (DEGs) from the white blood cells (WBCs) of five heterozygous FH patients and five healthy controls. The interaction between the DEGs was identified by applying the STRING tool and visualized using Cytoscape software. MCODE was used to determine the gene cluster in the interactive networks. The identified DEGs were subjected to the DAVID v6.8 webserver and ClueGo/CluePedia for functional annotation, such as gene ontology (GO) and enriched molecular pathway analysis of DEGs.

**Results:** We investigated the top 250 significant DEGs (*p*-value < 0.05; fold two change ≥ 1 or ≤ −1). The GO analysis of DEGs with significant differences revealed that they are involved in critical biological processes and molecular pathways, such as myeloid cell differentiation, peptidyl-lysine modification, signaling pathway of MyD88-dependent Toll-like receptor, and cell-cell adhesion. The analysis of enriched KEGG pathways revealed the association of the DEGs in ubiquitin-mediated proteolysis and cardiac muscle contraction. The genes involved in the molecular pathways were shown to be differentially regulated by either activating or inhibiting the genes that are essential for the canonical signaling pathways. Our study identified seven core genes (*UQCR11, UBE2N, ADD1, TLN1, IRAK3, LY96*, and *MAP3K1*) that are strongly linked to FH and lead to a higher risk of atherosclerosis.

**Conclusion:** We identified seven core genes that represent potential molecular biomarkers for the diagnosis of atherosclerosis and might serve as a platform for developing therapeutics against both FH and atherosclerosis. However, functional studies are further needed to validate their role in the pathogenesis of FH and atherosclerosis.

## Introduction

Atherosclerosis is a chronic immune-inflammatory disease that is characterized by the progressive accumulation of lipids in the intimal space of the atrial walls, which results in such complications as chronic low-grade inflammation, endothelial dysfunction, and oxidative stress. A high level of low-density lipoprotein (LDL) in plasma induces atherosclerosis. In contrast, a decreasing level of LDL cholesterol is associated with a decreased frequency of severe cardiovascular events (Silverman et al., 2016). Elevated levels of blood cholesterol are caused by a group of genetic defects known as familial hypercholesterolemia (FH). FH is one of the known genetic causes of premature cardiovascular disease due to prolonged exposure to elevated LDL, with a prevalence of ∼1:220 being observed (Abul-Husn et al., 2016; Wald et al., 2016; Alhababi and Zayed, 2018). Recent studies estimated the prevalence of heterozygous FH (HeFH) to be significantly higher (1/220-250) than initially reported (1/500) (Hopkins et al., 2011; Genest et al., 2014; Perez-Calahorra et al., 2019). However, the homozygous FH (HoFH) prevalence has been estimated to be 1 in 300,000–1,000,000 (Austin et al., 2004; Sjouke et al., 2015; de Ferranti et al., 2016; Akioyamen et al., 2017; Alhababi and Zayed, 2018; Alonso et al., 2018). The prevalence of FH is higher due to founder effect that estimates to be upto 1 in 50–67 in some populations like Lebanese, Ashkenazi Jews, French Canadians, Finns, Afrikaners, and Tunisians (Leitersdorf et al., 1990; Nanchen et al., 2015; Amor-Salamanca et al., 2017; Alonso et al., 2018). In the past, the term FH was used to refer to defects in the LDL receptor (Goldberg et al., 2011). Among FH patients, the clinical phenotypes are distinctly versatile, even in patients who share the same disease-causing mutation. This finding suggests that FH is not a single disease but is a multifaceted syndrome (Hartgers et al., 2015; Di Resta and Ferrari, 2018; Masana et al., 2019). HeFH is mainly caused by mutations that occur in such genes as *LDLR*, less frequently, mutations in *APOB* and *PCSK9* genes can be found in patients with phenotypic FH (Soutar and Naoumova, 2007; Gidding et al., 2015). Several studies reported that HoFH causes considerable premature ASCVD, and would result in early death if left untreated (males are at 50% risk, and females are at 30% risk) (Slack, 1969; Stone et al., 1974; Joseph et al., 2001; Naoumova et al., 2004; Vuorio et al., 2014). For the management of patients above 75 years of age with clinically evident atherosclerotic cardiovascular disease (ASCVD), the ACC/AHA standards endorse a moderate intensity (but not a high-intensity) statin (Vuorio et al., 2013, 2017; Stone et al., 2014). Microarray technology is a robust procedure that is widely used to compare genes that are differentially expressed in patients with different diseases. This technology is also beneficial in understanding gene association, mapping, expression, and linkage studies (Russo et al., 2003). However, studies that investigated the white blood cells (WBCs) transcriptome of patients with FH versus healthy controls are limited. Therefore, this study aimed to identify differentially expressed genes (DEGs), protein-protein interactions, and dysregulated pathways that might be involved in an increased risk of atherosclerosis due to FH.

## Materials and Methods

### Array Data Acquisition and Processing

The GEO database from NCBI^1^ was used to access the GSE13985 dataset that contains expression profiles by array. The datasets from various experiments are deposited in this database and enable users to download the gene expression profiles stored in GEO (Barrett et al., 2013). To seek GEO datasets for related gene expression profiles, we used the keywords “Familial Hypercholesterolemia” and “Microarray” and “Homo sapiens.” GSE13985 contains ten samples, including five patients with FH and five healthy control samples obtained with the help of platform GPL570 [HG-U133_Plus_2] Affymetrix Human Genome U133 Plus 2.0 Array (Režen et al., 2008). The five FH patients and five controls were matched by age, BMI, sex, and smoking status. The FH patients were free of clinical ASCVD. The Blood samples were provided from the ten samples, and RNA was extracted to be used for the array analysis. The gene expression data were downloaded from the public database, and in this study, there were no animal or human experiments assisted by any of the authors.

### Data Preprocessing and Identification of DEGs

**FIGURE 1:**
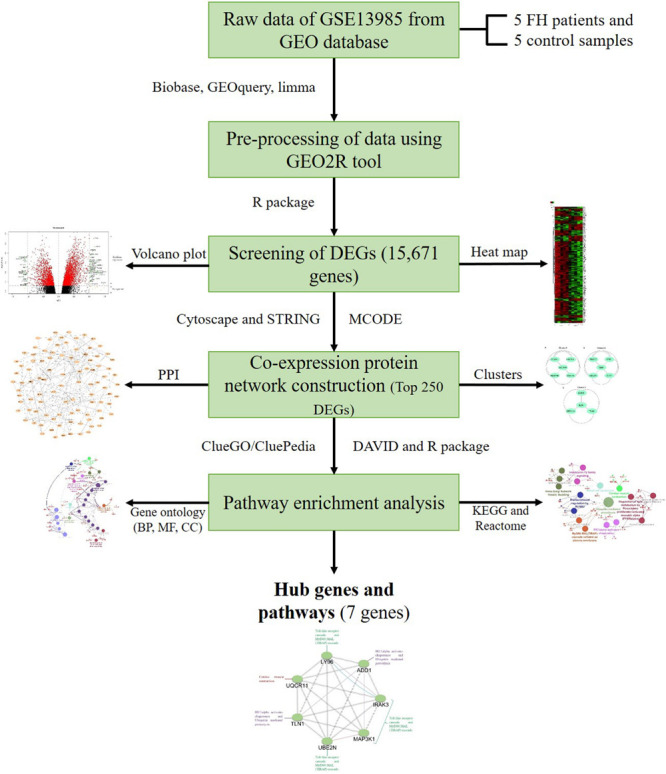
Outline of the workflow and expression data analysis in the present study using bioinformatic strategies.

With the help of powerful multiarray technology, the preliminary data from the dataset were made susceptible to the correction of background, quantile normalization, and log transition (Irizarry et al., 2003). Initial processing of the data involved altering specific gene symbols from probe IDs with the help of a Entrez’s Gene ID converter (Alibés et al., 2007). When the same gene contribution was observed in several samples, their mean value was determined and considered as the eventual level of gene expression. To examine the raw gene expression data, the online statistical tool GEO2R was utilized, and the tool incorporated the R/Bioconductor and Limma package v3.26.8 (Smyth, 2005; Barrett et al., 2013; Ritchie et al., 2015). The GEO2R inbuilt methods, such as *T*-test and Benjamini and Hochberg (false discovery rate), were used to calculate the *p*-value and FDR in order to determine the DEGs among patients with FH and controls (Aubert et al., 2004). We set the principal standards of | log (fold change) | > 1 and *p* < 0.05 to acquire DEGs that are significant from the dataset, whereas the upregulated DEGs were considered if the logFC ≥ 1 and logFC ≤ −1 for downregulated DEGs. The RStudio (v1.2.5019) and library Calibrate package were used to create the volcano plot. The subsequent DEGs were attained from a dataset, and further investigation was performed with selected DEGs. The heat map for the gene expression data was generated using a heat mapper webserver.^2^ The inbuilt average linkage clustering method was used to compute the hierarchical clustering, and the Euclidean algorithm was used for computing the distance between rows and columns (Babicki et al., 2016). The flowchart diagram for this study is represented in Figure 1.

### Establishment of PPI Networks and Module Analysis

We framed a PPI (protein-protein interaction) network by utilizing the STRING web-based tool (v11.0,^3^) to evaluate the relationship among the DEGs from the attained datasets (Szklarczyk et al., 2017, 2019). To eradicate the PPI interactions that are in consistent from the dataset, we fixed the cutoff standard to a confident interaction score ≥0.4. Therefore, we attained a strong PPI network. Then, we combined the outcomes from the STRING tool to Cytoscape software (v3.7.1,^4^) to conceptualize the PPI interactions among the statistically appropriate DEGs (Shannon et al., 2003). To recognize the intersected clusters from the attained PPI network, we exploited the Cytoscape plugin Molecular Complex Detection plugin (MCODE). The group (cluster) determining extremities were charted, such as Kappa score (K-core) fixed to five, Degree Cutoff fixed to two, Max. Depth fixed to 100, and Node score Cutoff fixed to 0.2, which constraints the cluster size for coexpressing networks (Bader and Hogue, 2003). Further, we utilized the GeneMANIA web server to perform the inter-relation analysis and predict the function of the identified seven potential DEGs (Warde-Farley et al., 2010; Franz et al., 2018).

### DAVID and ClueGO Enrichment Analysis

For functional annotation of GO and analysis of KEGG pathway enrichment, we used the web-based DAVID v6.8 tool.^5^ DAVID is a significant source for any functional evaluation of the high-throughput gene expression profiles (Huang et al., 2009a, b). The results from DAVID was further imported to GOplot in R Studio. The GOBubble and GOChord were used to visualize the functional enrichment of the top 250 DEGs, which facilitate the combination and integration of expression data with functional assessment results (Walter et al., 2015). For integrative analysis, we utilized both DAVID and ClueGO software to comprehensively observe the DEGs involved in the GO terms and pathways. The first DEGs from the GEO2R tool were exposed to ClueGO v2.5.5/CluePedia v1.5.5 to attain complete Gene Ontological terms (GO) and disease-related pathways from the DEG dataset. ClueGO syndicates KEGG or BioCarta pathways and GO, which delivers a fundamentally organized pathway network or GO from the DEG dataset (Bindea et al., 2009). Also, the study of molecular/biological function GO, and enrichment of pathways analysis was conducted for DEGs, and *p*-values < 0.05 were considered to be significant.

## Results

### Data Acquisition and Identification of DEGs

**FIGURE 2:**
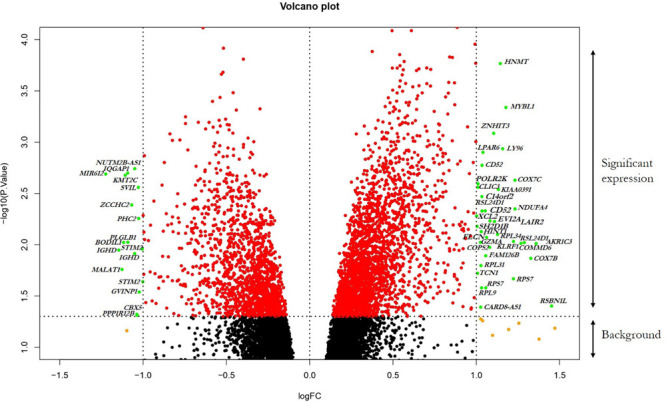
Visualization of DEGs volcano plots using R studio. The plot compared the DEGs between FH patients and controls from the dataset. The representations are as follows: *x*-axis, logFC; *y*-axis, -log10 of a *p*-value. The *p*-values < 0.05 are in red dots, and logFC ≥ 1 and logFC ≤ –1 are in yellow dots; the significant DEGs with both satisfying values are in green dots and indicated with gene names. Black dots indicate the remaining genes present in the array that were not significantly changed. The genes that are upregulated in the array are on the right panel, and downregulated ones are on the left panel of the plot.

**FIGURE 3:**
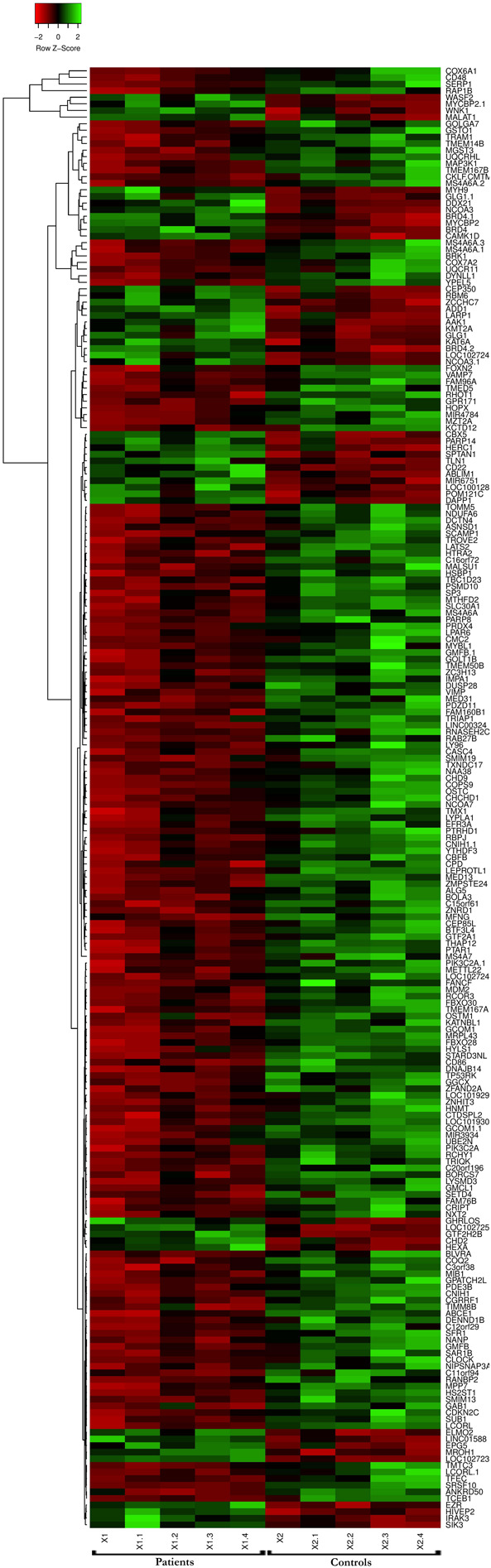
Microarray heat map and clustering based on hierarchy for the top 250 gene expression data from the datasets. *n* = 250 DEGs. Upregulated genes are represented in red, and downregulated genes are represented in green.

The GSE13985 dataset contained the gene expression profiles of five patient samples with FH, and five control groups (atherosclerotic markers in human blood - a study in patients with familial hypercholesterolemia) were obtained from the GEO database (Režen et al., 2008). The GPL570 platform (Affymetrix Human Genome U133 Plus 2.0 Array) was used in this work. Table 1 represents the original features of the patients and control samples involved in this study. By using the publicly available GEO2R tool, we identified the DEGs between FH patients and healthy controls according to the cutoff values of | log2FC| ≥ 1.0 and *p*-values < 0.05, which were calculated based on the inbuilt R/Bioconductor and limma package v3.26.8 from the GEO2R tool, the top 250 DEGs were accordingly identified. The R studio and library calibrate package was used to construct the volcano plot to compare the DEGs between FH patients and healthy controls. Figure 2 represents the volcano plot, and the significant genes with satisfying values (*p*-value < 0.05, logFC ≥ 1, and logFC ≤ −1) are labeled and shown in green dots. The top 250 DEGs identified between both groups were subjected to a heat mapper web server to determine the expression level of the genes. As a result, clustering based on hierarchy and heat maps was generated and is depicted in Figure 3.

**TABLE 1:** Information on patients and controls primary features in GSE13985 from the GEO database.

Group	Accession	Title	Organism	Gender	Age	Disease State	Tissue
Patient	GSM351336	Patient 1	Homo sapiens	Male	33 years	Heterozygous familial hypercholesterolemia	Blood
	GSM351337	Patient 2	Homo sapiens	Male	33 years	Heterozygous familial hypercholesterolemia	Blood
	GSM351338	Patient 3	Homo sapiens	Male	46 years	Heterozygous familial hypercholesterolemia	Blood
	GSM351339	Patient 4	Homo sapiens	Male	22 years	Heterozygous familial hypercholesterolemia	Blood
	GSM351340	Patient 5	Homo sapiens	Male	35 years	Heterozygous familial hypercholesterolemia	Blood
Control	GSM351341	Control 1	Homo sapiens	Male	35 years	None	Blood
	GSM351342	Control 2	Homo sapiens	Male	37 years	None	Blood
	GSM351343	Control 3	Homo sapiens	Male	22 years	None	Blood
	GSM351344	Control 4	Homo sapiens	Male	45 years	None	Blood
	GSM351345	Control 5	Homo sapiens	Male	33 years	None	Blood

### Establishment of PPI Networks and Module Analysis

**FIGURE 4:**
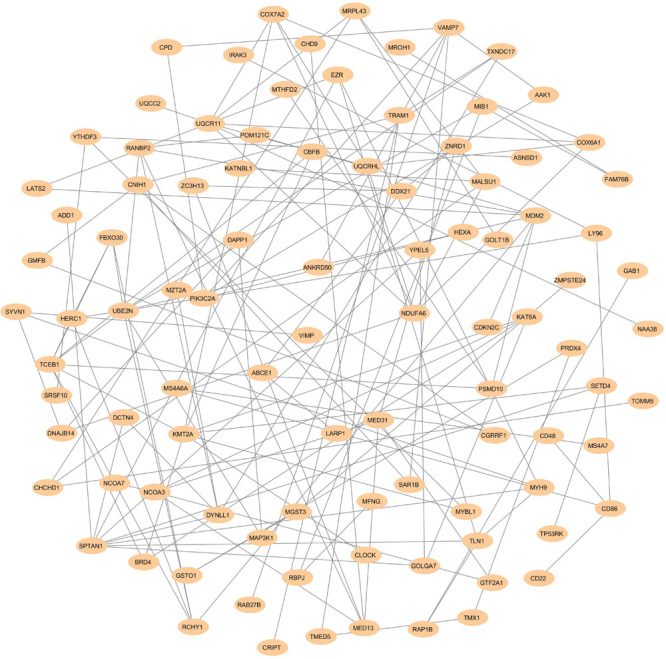
PPI networks show the interaction of DEGs from the GSE13985 dataset. The nodes and edges are retrieved from the STRING tool and plotted using Cytoscape software. The nodes are displayed as Navajo white, while edges are shown as gray.

**FIGURE 5:**
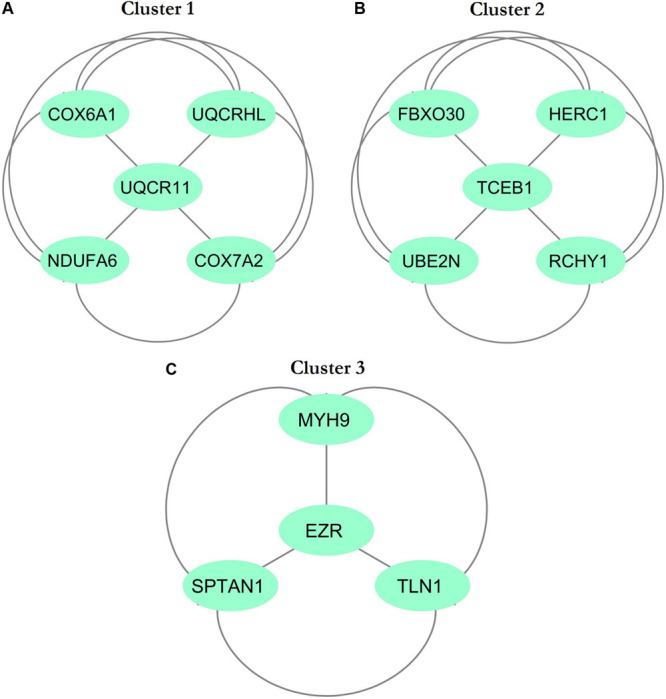
Top three cluster subnetworks were identified from the PPI network with the help of Cytoscape using the MCODE plugin with a cluster score above three. The aquamarine (ellipse) color indicates the nodes, while the edges are in gray. **(A)** Cluster 1; **(B)** Cluster 2; **(C)** Cluster 3.

The physical and functional associations among the proteins of DEGs of FH were assessed using the STRING tool. The minimum required interaction score was set to the confidence of 0.4. Simple tabular text output was generated from STRING. The interaction among the query proteins was further visualized using Cytoscape v3.7.1. Figure 4 represents the network with 96 nodes and 134 edges of PPI. The nodes denote the number of proteins while edges and their interactions. The Cytoscape plugin MCODE v1.5.1 interpreted the closely interlinked regions from the network of proteins in the form of clusters. The top three clusters that are significant from the PPI network with MCODE scores of 5, 5, and 4 were preferred. These clusters are represented graphically in Figure 5. Cluster 1 was derived from node *UQCR11* (Figure 5A). Cluster 2 and Cluster 3 were derived from nodes *TCEB1* and *EZR*, respectively (Figures 5B,C). The tabular column represents the detailed MCODE clusters of interlinked regions with their cluster number, MCODE scores, node IDs, node numbers, and edge numbers (Table 2).

**TABLE 2:** The most interlinked regions are clustered from the DEGs of GSE13985 dataset using MCODE.

Cluster	Score (Density x No. of nodes)	Nodes	Edges	Node IDs
1	5	5	10	*COX7A2, NDUFA6, UQCR11, UQCRHL, COX6A1*
2	5	5	10	*FBXO30, HERC1, TCEB1, UBE2N, RCHY1*
3	4	4	6	*EZR, SPTAN1, TLN1, MYH9*

### DAVID Enrichment Analysis

**FIGURE 6:**
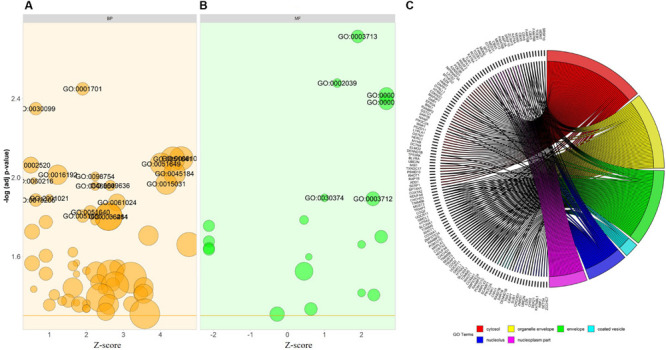
Bubble plot depicts the **(A)** biological process (BP-in orange), and **(B)** molecular function (MF-in green) of the GO terms from DAVID. The –log *p*-value allocated to the y-axis and a *Z*-score to *x*-axis. The region of bubbles are significantly proportional to the number of DEGs in a given GO term. The threshold implies the *p*-value standardized to 0.05 (orange line in BP and MF). **(C)** The association between the selected DEGs; their respective cellular component (CC) and GO terms, along with the gene’s log2FC are presented in GOChord plot. The plot’s left half showed the DEGs that involved in different cellular components and the right half displays the GO terms of cellular components in various colors. The colored bands connected a gene to a specific term GO.

For the functional annotation of DEGs, the DAVID v6.8 online server was used. To ascertain the KEGG pathway-enriched genes and the potential GO (Gene Ontology) classification, terms approximating biological process, molecular functions, and signaling pathways concerning KEGG pathways were used. The modified Fisher exact *p*-value (EASE score) ≤ 0.05 and FDR < 0.05 are considered strongly enriched. By analyzing BP, we found that the DEGs from the complex PPI network were enriched in myeloid cell differentiation (GO: 0030099), intracellular transport (GO: 0046907), negative regulation of response to DNA damage stimulus (GO: 2001021), peptidyl-lysine modification (GO: 0018205), negative regulation of signal transduction by p53 class mediator (GO: 1901797), and MyD88-dependent toll-like receptor signaling pathway (GO: 0002755). The Gene Ontology MF analysis revealed the involvement of DEGs in antioxidant activity (GO: 0016209), p53 binding (GO: 0002039), thyroid hormone receptor binding (GO: 0046966), cadherin binding involved in cell-cell adhesion (GO: 0098641), transcription factor activity, and transcription factor binding (GO: 0000989). In addition, we utilized the DAVID online method to classify the DEGs entailed in the different biological pathways based on the KEGG reference database (*p* < 0.05; FDR < 0.05). The KEGG pathway enrichment analysis revealed the association of the DEGs in ubiquitin-mediated proteolysis (hsa04120) and cardiac muscle contraction (hsa04260). The annotated results for the following terms were tabulated (Table 3). The GOBubble plots were constructed for the BP and MF of the top 250 DEGs from the dataset (Figures 6A,B), whereas the enriched cellular components of the identified DEGs were plotted with GOChord (Figure 6C).

**TABLE 3:** Gene ontology (GO) terms such as biological process, molecular functions, and KEGG pathways of DEGs that are associated with familial hypercholesterolemia from DAVID.

Category	Term	Count	%	*p*-value	Fold enrichment	FDR
GOTERM_BP_FAT	GO:0030099∼Myeloid cell differentiation	10	4.9	4.9E-3	3.1	8.4E0
GOTERM_BP_FAT	GO:0046907∼Intracellular transport	24	11.8	2.3E-2	1.6	3.4E1
GOTERM_BP_FAT	GO:2001021∼Negative regulation of response to DNA damage stimulus	4	2.0	1.3E-2	8.1	2.1E1
GOTERM_BP_FAT	GO:0018205∼Peptidyl-lysine modification	10	4.9	1.4E-2	2.6	2.2E1
GOTERM_BP_FAT	GO:1901797∼Negative regulation of signal transduction by p53 class mediator	3	1.5	2.8E-2	11.4	4.0E1
GOTERM_BP_FAT	GO:0002755∼MyD88-dependent toll-like receptor signaling pathway	3	1.5	3.8E-2	9.7	5.0E1
GOTERM_MF_FAT	GO:0016209∼Antioxidant activity	4	2.0	3.9E-2	5.3	4.3E1
GOTERM_MF_FAT	GO:0002039∼p53 binding	5	2.5	3.4E-3	8.0	4.8E0
GOTERM_MF_FAT	GO:0046966∼Thyroid hormone receptor binding	3	1.5	2.6E-2	11.9	3.1E1
GOTERM_MF_FAT	GO:0098641∼Cadherin binding involved in cell-cell adhesion	8	3.9	1.8E-2	3.0	2.3E1
GOTERM_MF_FAT	GO:0000989∼Transcription factor activity, transcription factor binding	14	6.9	4.1E-3	2.5	5.7E0
KEGG_PATHWAY	hsa04120:Ubiquitin mediated proteolysis	6	3.0	1.3E-2	4.2	1.4E1
KEGG_PATHWAY	hsa04260:Cardiac muscle contraction	4	2.0	4.2E-2	5.1	3.9E1

### ClueGO/CluePedia Enrichment Analysis

**FIGURE 7:**
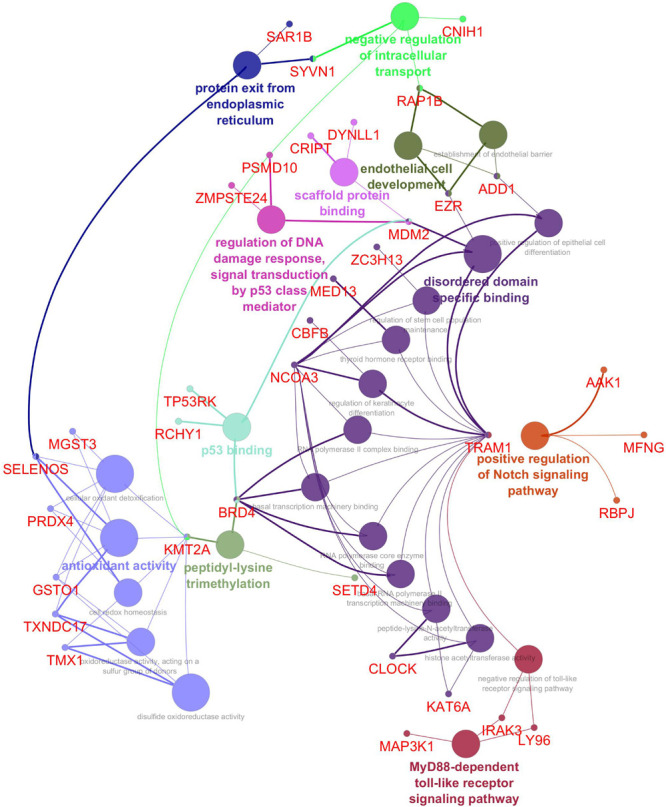
Enrichment by Gene Ontology (GO) terms was visualized using the ClueGO/CluePedia plugin from Cytoscape. Vital molecular functions (MF) and biological processes (BP) involved in the DEGs are shown with the specific gene interactions. The MF and BP enrichment analyses are inferred from the 250 top DEGs PPI network. The connectivity of the GO terms network described by functional nodes and edges that are shared between the DEGs with a kappa score of 0.4. The enrichment shows only significant GO terms (*p*-value ≤ 0.05). The values of *p* ≤ 0.05 indicate the node size. The node color code indicates the specific functional class that they are involved in. The color represents various molecular function and biological process involved in the enrichment analysis. The bold fonts indicate the most important functional GO terms that define the names of MF and BP of each group. The names of the DEGs involved in each group are displayed in red font.

**FIGURE 8:**
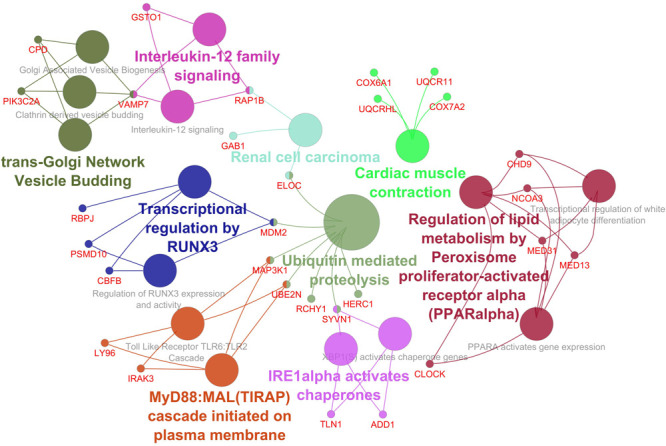
Enrichment by pathway terms is visualized using the ClueGo/CluePedia plugin from Cytoscape. The plugin delivers a comprehensive enrichment analysis for DEGs, including KEGG and REACTOME pathways. The connectivity of the pathways in the network described by functional nodes and edges that are shared between the DEGs with a kappa score of 0.4. The enrichment shows only significant pathways (*p*-value ≤ 0.05). The values of *p* ≤ 0.05 indicate the node size. The node color code indicates the specific functional class that they are involved in. The color represents various molecular pathways involved in the enrichment analysis of the identified DEGs. The bold fonts indicate the most critical functional pathways that define the names of the signaling pathway of each group. The names of the DEGs involved in each group are displayed in red font.

The Cytoscape plugin ClueGO/CluePedia was used to study the functional enrichment of the DEGs from the dataset. ClueGo helped visualize the GO terms of the identified PPI complex network. The MF and BF terms of the GO functional enrichment analysis of the complex PPI network are depicted in Figure 7. The statistical options for ClueGO enrichment analysis were set based on a hypergeometric test that is two-sided with *p* ≤ 0.05, Benjamini-Hochberg correction, and kappa score ≥ 0.4 as a primary criterion. The BF and MF of DEGs from the complex PPI network were predominantly enriched in the negative regulation of intracellular transport (GO: 0032387), endothelial cell development (GO: 0001885), scaffold protein binding (GO: 0097110), regulation of DNA damage response and signal transduction by p53 class mediator (GO: 0043516), p53 binding (GO: 0002039), peptidyl-lysine trimethylation (GO: 0018023), antioxidant activity (GO: 0016209), positive regulation of the Notch signaling pathway (GO: 0045747), and MyD88-dependent Toll-like receptor signaling pathway (GO: 0002755) (Figure 7). The KEGG and REACTOME pathway analysis from ClueGO showed that many DEGs were significantly enriched in cardiac muscle contraction (KEGG: 04260), regulation of lipid metabolism by peroxisome proliferator-activated receptor alpha (PPAR alpha) (R-HAS: 400206), transcriptional regulation by *RUNX3* (R-HAS: 8878159), ubiquitin-mediated proteolysis (KEGG: 04120), renal cell carcinoma (KEGG: 05211), MyD88: MAL (TIRAP) cascade initiated on plasma membrane (R-HAS: 166058), and IRE1 alpha-activated chaperones (R-HAS: 381070) (Figure 8). Taken together, the results from ClueGO enrichment clearly illustrate that the DEGs change the metabolic behavior of the signaling pathways and are closely linked to FH, contributing to the progression of such complications as coronary artery disease and cardiovascular disease, which may lead to atherosclerosis. Additionally, the dysregulated pathways identified by our bioinformatics enrichment analysis could play important roles in FH pathogenesis. However, functional validations are needed to test our bioinformatics findings.

## Discussion

In the present study, we investigated the DEGs between five patients with FH and five healthy controls with the GEO ID of GSE13985 (Režen et al., 2008). We examined a total of 13,057 DEGs, and the top 250 significant DEGs were considered for further studies (Supplementary Table S1). We found the top three significant clusters that are distinguished by high scores and closely interconnected regions from the networks of PPI (Figures 5A–C). Screening the gene cluster could help in determining the crucial genes and their interactions and how they are associated with the pathogenesis and progression of atherosclerosis. The clusters retrieved from MCODE were often shown in the PPI network mechanisms, and representation of clusters is crucial for a functional and comprehensive understanding of network properties (Krogan et al., 2006; Rahman et al., 2013). The densely interconnected nodes and the less connected vertices of the PPI network were weighed using the core clustering coefficient of the MCODE plugin. After computation, an algorithm examined the weighted graph to isolate the densely connected regions, which is considered as clusters and represents the molecular complexes that formed with DEGs (Sharan et al., 2007). From Table 2, we found three seed nodes, namely, the *UQCR11*, *TCEB1*, and *EZR* genes that might have been involved in the differential regulation of the pathway.

To explore the involvement of the 250 identified DEGs in BP, MF, and molecular pathways of FH, we used the built GO and KEGG enrichment to determine the functional annotation of these genes. We found that these DEGs were primarily enriched in myeloid cell differentiation, intracellular transport, negative regulation of response to DNA damage stimulus, peptidyl-lysine modification, negative regulation of signal transduction by p53 class mediator, and the MyD88-dependent Toll-like receptor signaling pathway. The analysis of MF from GO showed that the DEGs were significantly enriched in antioxidant activity, p53 binding, thyroid hormone receptor binding, cadherin binding involved in cell-cell adhesion, and transcription factor activity. Similarly, the analysis of KEGG pathway enrichment showed that the DEGs are involved in ubiquitin-mediated proteolysis and cardiac muscle contraction (Table 3). Interestingly, MyD88-mediated signaling plays a prominent role in the development of human atherosclerosis and matrix degradation (Monaco et al., 2009). In line with this finding, Yu et al. (2014) found that MyD88-deficient myeloid cells are involved in the inhibition of macrophage recruitment to adipose tissue and result in atherosclerosis and diet-induced systemic inflammation (Yu et al., 2014). In this context, our study identified the DEGs involved in the MyD88 signaling pathway, such as *IRAK3* (interleukin 1 receptor-associated kinase 3), *LY96* (lymphocyte antigen 96), and *MAP3K1* (mitogen-activated protein kinase 1). Among these genes, *LY96* and *MAP3K1* were significantly downregulated in the FH patients, whereas *IRAK3* was upregulated compared to the healthy controls (Supplementary Figure S1). *IRAK3* prevents *IRAK1* and *IRAK4* from dissociating from *MyD88* and inhibits the formation of IRAK-TRAF6 complexes (Lyu et al., 2018; Yu and Feng, 2018). The increased expression of *IRAK3*, as shown in Supplementary Figure S1A, typically coveys the dysregulation of MyD88 and the TLR cascade via *IRAK3* expression. Similarly, the receptor complex resulting from the combination of *LY96* and Toll-like receptor 4 (*TLR4*) ectodomain mediates transduction of the lipopolysaccharide (LPS) signal across the cell membrane (Gruber et al., 2004). A recent study found that *LY96* can bind to cholesterol (Choi et al., 2016), and *TLR4* activation involves agLDL, the predominant form of LDL found in atherosclerotic plaques (Singh et al., 2020). Thus, our examined DEGs are reliable with involvement in atherosclerosis-causing pathways.

To further refine the biological process, molecular functions, and pathways defined from the analysis of DAVID-GO terms (Figure 6), KEGG, and STRING, we implemented the ClueGO plugin from Cytoscape, an improved interpretation for biological terms, such as GO and KEGG pathway analysis/BioCarta, and constructed a functionally arranged network of terms GO/pathway. The plugin also helps to visualize the networks that are functionally grouped from more massive gene clusters (Bindea et al., 2009). To acquire a detailed picture of the DEGs involved in atherosclerosis, we utilized the ClueGO plugin to distinguish the molecular pathways that are differentially regulated and their significant gene interactions depending on the *p*-values and kappa statistics.

Among the enriched biological process and molecular pathways, we identified that ubiquitin-mediated proteolysis, cardiac muscle contraction, MyD88-dependent Toll-like receptor signaling pathway, and IRE1 alpha-activated chaperones were considered dysregulated and are significant to atherosclerosis progression in FH patients based on kappa statistics and *p*-values (Figures 7, 8). Additionally, we also identified the genes that are significant in the dysregulated molecular pathways and involved in FH progression. In line with this finding, *UQCR11, UBE2N, ADD1, TLN1, IRAK3, LY96*, and *MAP3K1* were found to be associated with the risk of Atherosclerosis in FH patients.

This study identified DEGs that are involved in ubiquitin-mediated proteolysis, IRE1 alpha-activated chaperones, and cardiac muscle contraction, such as *UQCR11, UBE2N, TLN1*, and *ADD1*, respectively. As shown in Supplementary Figure S2, a significant gene expression level was shown between the FH patients and healthy control samples. In this study, the expression levels of the *UQCR11* and *UBE2N* genes were significantly reduced, and the expression levels of the *TLN1* and *ADD1* genes were notably increased in the FH patients compared with the healthy controls. Our identified novel DEGs from the dataset indeed have high consistency with the risk of atherosclerosis and are involved in the dysregulation of such pathways as ubiquitin-mediated proteolysis, IRE1 alpha-activated chaperones, and cardiac muscle contraction. For instance, the transfer of ubiquitin from UBE2N to LDLR is required for its lysosomal degradation. The reduced gene expression level of *UBE2N* leads to skipping this event and might result in the accumulation of LDLR in the lysosome (Zhang et al., 2012).

Additionally, the polymorphism (p.G460W) present in ADD1 has reportedly contributed to the increased risk factor for coronary heart disease (Morrison et al., 2002). Our study clearly showed the increased expression of *ADD1* from the FH patient dataset, which could alter the casual signaling of IRE1 alpha-activated chaperones (Supplementary Figure S2D). *TLN1* functions as a molecular scaffolding protein and can contribute to the signaling of adhesion through its binding partners, translating mechanical signals into chemical signals (Hytönen and Wehrle-Haller, 2014). The *TLN1* expression level in atherosclerotic plaques is significantly reduced, and it plays a central role in cell adhesion, indicating that tissue disintegration in atherosclerosis may be partly induced by *TLN1* downregulation, leading to cell-ECM interaction loosening and tissue reorganization (Essen et al., 2016). However, our dataset showed increased expression of *TLN1*, which might differentially regulate and activate IRE1alpha chaperones. Yet, recent research has identified that proatherogenic gene expression is regulated by *IRE1*, which includes several essential chemokines and cytokines (Tufanli et al., 2017).

**FIGURE 9:**
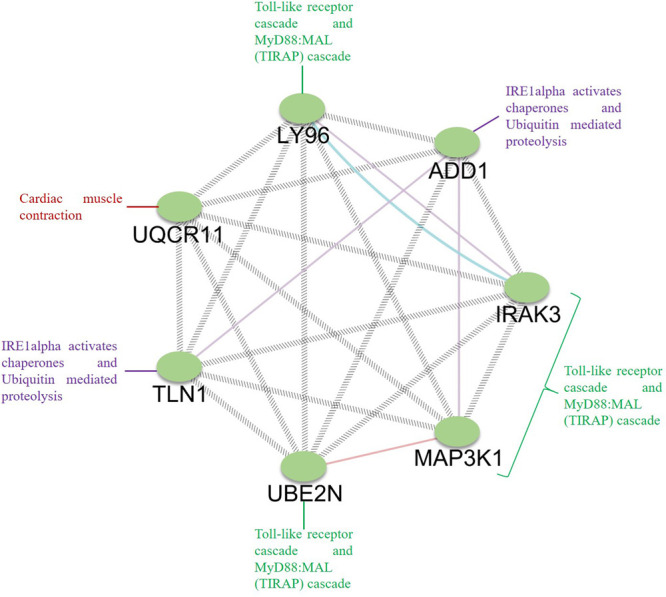
Analysis of the interrelation between the identified core genes. GeneMANIA was used to map the network, and Cytoscape was used for network visualization. The genes *LY96, IRAK3, MAP3K1*, and *UBE2N* are involved in the Toll-like receptor cascade and MyD88:MAL (TIRAP) cascade; *ADD1* and *TLN1* are involved in ubiquitin-mediated proteolysis, IRE1alpha activates chaperones, and *UQCR11* is involved in cardiac muscle contraction.

To establish the interrelationship between the core genes *UQCR11*, *UBE2N*, *ADD1*, *TLN1*, *IRAK3*, *LY96*, and *MAP3K1*, this interrelationship will help in understanding the coexpression and molecular pathways that pertain to FH. It is essential to comprehend the interactions between the core genes since these genes dysregulate the general molecular pathways in FH patients (Kumar et al., 2019; Udhaya Kumar et al., 2020). The differentially expressed genes might be responsible for the clinical phenotypes of the patients and the progression of atherosclerosis. We found that the Toll-like receptor and MyD88:MAL (TIRAP) cascades are activated by such DEGs as *LY96, IRAK3, MAP3K1*, and *UBE2N* (Monaco et al., 2009; Yu et al., 2014). Similarly, *UQCR11* is directly involved in cardiac muscle contraction and has a physical interaction between *LY96, TLN1*, and *UBE2N*. The *TLN1* and *ADD1* genes directly associated with IRE1alpha-activated chaperones and ubiquitin-mediated proteolysis (Figure 9). Taken together, our observed findings suggest that the involvement of core genes related to the risk of atherosclerosis might be a critical metric in ubiquitin-mediated proteolysis, Toll-like receptor, and MyD88: MAL (*TIRAP*) cascades and a beneficial tool for diagnosis and targeted therapy. In addition, the FH patients were free of clinical ASCVD, and the patient 2 and 5 did not receive lipid-lowering treatment, whereas the patient 1, 3, and 4 received lovastatin, simvastatin, and atorvastatin, respectively. This drug is also given to the patients as primary and secondary prevention that develops the risk of ASCVD and for those who already developed ASCVD, respectively (Taylor et al., 2013; Vuorio et al., 2013). With the help of a clinical diagnostic strategy, approximately 50% of patients are identified in FH, which is a cost-effective process. Indeed, a diagnosis by screening the cascades is a systematic and useful tool for diagnosing FH patients before the development of Atherosclerosis (Marks et al., 2002; Leren et al., 2008).

In general, FH can be progressed through many cellular and molecular mechanisms, and pathways. A recent study claimed that the *COX7B* is a potential novel gene target for FH treatment (Li et al., 2015); however, authors did not provide supportive evidence for metabolic network and pathway links between the Atherosclerosis and FH. Later, a similar study claimed that the *RPL17* and ribosome protein-related genes might increase the atherosclerotic risk. Although the study mentioned that these genes are downregulated in the FH patient’s blood cells, they are significantly upregulated in the reported dataset. In addition, they claimed that cytochrome-c oxidase genes could contribute to atherosclerosis development, yet there was no substantial evidence provided (Wang and Zhao, 2016). The study conducted by Smolock et al. (2012) suggested that RPL17 acts as an inhibitor for vascular smooth muscle cells (VSMC) and might be a therapeutic target to limit the thickening of carotid intima (Smolock et al., 2012). However, increased expression of *RPL17* showed to inhibit the VSMC, reducing the progression of atherosclerosis, which could be a regular event that occurs in FH patients.

Interestingly, our functional enrichment analysis did not capture any biological/molecular functions related to inflammatory responses, as some patients with FH had shown inflammatory responses due to increased expression level of the molecules associated with tumor necrosis factor (TNF) (Fadini et al., 2014; Holven et al., 2014; Escate et al., 2018). The possible reason for this might be that the patients with FH in our study do not have any phenotypes characteristics to inflammation; consistent with the nature of FH, as it is a frequent disease (1/220-250) with a high variation in phenotypic expression associated with ASCVD (Alhababi and Zayed, 2018; Perez-Calahorra et al., 2019). Also, in era of personalized medicine, it is significant to identify the possible targeted therapy using advanced omics technologies (Prodan Žitnik et al., 2018).

Our study is the first to identify the association of core DEGs to the dysregulated pathways in FH patients. Our study recommends to screen WBCs from patients with FH to determine the metabolic and genetic factors that may help in identifying potential cardiovascular risks and might provide better diagnosis and improved therapy for the disease. The limitation of the present research is the small patient groups; therefore, investigating a larger sample size in different populations would help to confirm our data. The limitations of this study include; first, FH patients were free of clinical ASCVD and not possible to know if they have subclinical atherosclerosis by imaging. Second, the number is too small; therefore, it is important to study a larger cohort of patients that have clinical ASCVD that are compared with ones with no clinical ASCVD. Finally, no data were available for the LDL-C or TC of the FH patients in the GSE13985 dataset.

## Conclusion

Most FH patients do not exhibit atherosclerotic symptoms in clinical diagnostic procedures. The findings using a transcriptome analysis from WBCs of FH patients and healthy controls to identify atherosclerotic markers are highly limited. Overall, our systematic interpretation demonstrated an essential role of DEGs and their essential role in the occurrence, development of FH, and increased risk of atherosclerosis. Our study identified a total of 250 genes that are differentially expressed and seven essential genes that are associated with FH patients compared to healthy controls. The study of expression from WBCs and the association with DEGs may help to elucidate the role played by these DEGs in FH progression and the development of atherosclerosis. Finally, we identified seven novel potential target genes (*UQCR11, UBE2N, ADD1, TLN1, IRAK3, LY96*, and *MAP3K1*), which might be valid targets for therapeutic development for FH, and might be used as diagnostic biomarkers for FH patients and prognostic indicators for atherosclerosis using WBCs from FH patients; however, functional studies are needed to validate their proposed role in FH and atherosclerosis.

## Data Availability Statement

The GEO database from NCBI (Gene Expression Omnibus database, https://www.ncbi.nlm.nih.gov/geo/) was used to access the GSE13985 dataset.

## Author Contributions

SU, DT, HZ, and CG were involved in the design of the study and the acquisition, analysis, and interpretation of the data. SU, DT, RB, SS, RM, MS, HZ, and CG were involved in the interpretation of the data and drafting the manuscript. CG and HZ supervised the entire study and were involved in study design, the acquisition, analysis, and understanding of the data, and drafting the manuscript. All authors contributed to the article and approved the submitted version.

## Conflict of Interest

The authors declare that the research was conducted in the absence of any commercial or financial relationships that could be construed as a potential conflict of interest.

## Footnotes

1 https://www.ncbi.nlm.nih.gov/geo/

2 http://heatmapper.ca/expression/

3 http://www.string-db.org/

4 http://www.cytoscape.org/

5 https://david.ncifcrf.gov/

## Abbreviations

BP: biological process
FDR: false discovery rate
GO: gene ontology
KEGG: kyoto encyclopedia of genes and genomes
MF: molecular function.
