# Supplementary material for: Analysis of Differentially Expressed Genes and Molecular Pathways in Familial Hypercholesterolemia Involved in Atherosclerosis: A Systematic and Bioinformatics Approach
Source: Front Genet. 2020 Jul 15;11:734. doi: 10.3389/fgene.2020.00734 (PMC7373787; doi:10.3389/fgene.2020.00734)
Supplement: Supplementary file 1 [file Table_1.DOCX]

**Supplementary table 1. The list of top 250 DEGs identified from the dataset of two different groups.**

| Gene symbol | p-value | Log2FC |
| --- | --- | --- |
| *MYBL1* | 0.0004586 | 1.178 |
| *LY96* | 0.0011591 | 1.158 |
| *HNMT* | 0.0001711 | 1.144 |
| *ZNHIT3* | 0.0008193 | 1.104 |
| *LPAR6* | 0.0012557 | 1.04 |
| *HOPX* | 0.0001698 | 0.997 |
| *LOC101929356///ARL5A* | 0.0009879 | 0.997 |
| *GOLT1B* | 0.0001108 | 0.993 |
| *MS4A7* | 0.0015787 | 0.982 |
| *UQCR11* | 0.0007088 | 0.948 |
| *PTRHD1* | 0.0015295 | 0.904 |
| *PARP8* | 0.0000759 | 0.885 |
| *DNAJB14* | 0.0004316 | 0.871 |
| *SLC30A1* | 0.0002638 | 0.869 |
| *GGCX* | 0.0001493 | 0.857 |
| *TMEM50B* | 0.0009685 | 0.856 |
| *MS4A6A* | 0.0001476 | 0.84 |
| *MS4A6A* | 0.0002613 | 0.835 |
| *PRDX4* | 0.0015003 | 0.834 |
| *MIB1* | 0.0003326 | 0.832 |
| *RAB27B* | 0.0000393 | 0.83 |
| *CHD9* | 0.0005316 | 0.829 |
| *FBXO30* | 0.0003618 | 0.816 |
| *COX7A2* | 0.001293 | 0.815 |
| *NXT2* | 0.0008599 | 0.8 |
| *DENND1B* | 0.0012792 | 0.8 |
| *TMEM167A* | 0.0013786 | 0.8 |
| *CMC2* | 0.0015754 | 0.798 |
| *RCOR3* | 0.0000569 | 0.787 |
| *GMFB* | 0.0011796 | 0.787 |
| *NIPSNAP3A* | 0.0011605 | 0.786 |
| *LCORL* | 0.0000317 | 0.783 |
| *YTHDF3* | 0.0006772 | 0.78 |
| *MTHFD2* | 0.0009424 | 0.779 |
| *MS4A6A* | 0.0006643 | 0.774 |
| *C12orf29* | 0.0007888 | 0.774 |
| *CD48* | 0.0010869 | 0.771 |
| *UBE2N* | 0.001338 | 0.766 |
| *EFR3A* | 0.0015768 | 0.749 |
| *OSTM1* | 0.000501 | 0.747 |
| *NAA38* | 0.0015826 | 0.747 |
| *LYSMD3* | 0.0010505 | 0.744 |
| *GPR171* | 0.0000182 | 0.743 |
| *KCTD12* | 0.0014456 | 0.741 |
| *KATNBL1* | 0.0001953 | 0.727 |
| *COPS9* | 0.0002834 | 0.725 |
| *FAM76B* | 0.0010636 | 0.721 |
| *FAM96A* | 0.0009675 | 0.711 |
| *TFEC* | 0.0011036 | 0.705 |
| *OSTC* | 0.0002686 | 0.703 |
| *TMED5* | 0.000781 | 0.703 |
| *GPATCH2L* | 0.0001888 | 0.702 |
| *LYPLA1* | 0.0007008 | 0.694 |
| *MS4A6A* | 0.0003305 | 0.675 |
| *CHCHD1* | 0.0014477 | 0.672 |
| *MDM2* | 0.0014854 | 0.669 |
| *ZFAND2A* | 0.00087 | 0.662 |
| *TRIQK* | 0.0001928 | 0.656 |
| *GCOM1///POLR2M* | 0.0002813 | 0.656 |
| *GCOM1///POLR2M* | 0.0015692 | 0.655 |
| *TMX1* | 0.0013355 | 0.65 |
| *CKLF-CMTM1///CKLF* | 0.0003526 | 0.649 |
| *CPD* | 0.0008641 | 0.645 |
| *LOC101930404* | 0.0000603 | 0.644 |
| *PTAR1* | 0.0003784 | 0.643 |
| *CRIPT* | 0.0004793 | 0.642 |
| *RCHY1* | 0.0002003 | 0.641 |
| *FAM160B1* | 0.0006159 | 0.634 |
| *C3orf38* | 0.0009801 | 0.631 |
| *SAR1B* | 0.0012586 | 0.63 |
| *TOMM5* | 0.0009755 | 0.622 |
| *NDUFA6* | 0.0002885 | 0.619 |
| *NCOA7* | 0.0009043 | 0.619 |
| *TROVE2* | 0.0006511 | 0.617 |
| *COX6A1* | 0.001426 | 0.614 |
| *STARD3NL* | 0.0000817 | 0.61 |
| *HS2ST1* | 0.0000495 | 0.602 |
| *SUB1* | 0.0004079 | 0.599 |
| *CLOCK* | 0.0012237 | 0.596 |
| *SFR1* | 0.0004364 | 0.593 |
| *FOXN2* | 0.0011931 | 0.593 |
| *BTF3L4* | 0.0006603 | 0.591 |
| *CEP85L* | 0.0008355 | 0.59 |
| *TMEM14B///TMEM14C* | 0.0005523 | 0.587 |
| *MIR3934///UQCC2* | 0.0014817 | 0.587 |
| *CDKN2C* | 0.0012053 | 0.58 |
| *MIR4784///MZT2A///MZT2B* | 0.0004106 | 0.579 |
| *CNIH1* | 0.0002676 | 0.576 |
| *DCTN4* | 0.0006454 | 0.576 |
| *ASNSD1* | 0.0005759 | 0.573 |
| *TRAM1* | 0.0012767 | 0.569 |
| *MRPL43* | 0.0001634 | 0.567 |
| *CBFB* | 0.0009191 | 0.566 |
| *GMCL1* | 0.0001968 | 0.562 |
| *MGST3* | 0.0014658 | 0.561 |
| *MZT2A///MZT2B///PHGDH* | 0.0003187 | 0.558 |
| *NANP* | 0.0006418 | 0.558 |
| *TP53RK* | 0.0002204 | 0.556 |
| *LATS2* | 0.0006529 | 0.556 |
| *RBPJ* | 0.000671 | 0.546 |
| *ZMPSTE24* | 0.0002519 | 0.544 |
| *PSMD10* | 0.0001795 | 0.542 |
| *SP3* | 0.0014436 | 0.542 |
| *SMIM13* | 0.00014 | 0.54 |
| *TXNDC17* | 0.0006781 | 0.54 |
| *TBC1D23* | 0.0002881 | 0.539 |
| *GMFB* | 0.000495 | 0.538 |
| *BRK1* | 0.0015549 | 0.536 |
| *CNIH1* | 0.0000659 | 0.535 |
| *RNASEH2C* | 0.0013825 | 0.535 |
| *MAP3K1* | 0.0009808 | 0.534 |
| *CTDSPL2* | 0.0005846 | 0.531 |
| *SETD4* | 0.0008914 | 0.531 |
| *ALG5* | 0.0009789 | 0.526 |
| *CD86* | 0.0001893 | 0.525 |
| *VAMP7* | 0.0003311 | 0.525 |
| *GSTO1* | 0.0008253 | 0.525 |
| *METTL22* | 0.0003977 | 0.522 |
| *LCORL* | 0.0011549 | 0.519 |
| *MPP7* | 0.0010653 | 0.515 |
| *UQCRHL///UQCRH* | 0.0014196 | 0.515 |
| *VIMP* | 0.0004759 | 0.514 |
| *MED31* | 0.0004543 | 0.505 |
| *FBXO28* | 0.0009988 | 0.505 |
| *TMTC3* | 0.0011913 | 0.503 |
| *PDE3B* | 0.0005014 | 0.501 |
| *C16orf72* | 0.0003108 | 0.498 |
| *RHOT1* | 0.0001894 | 0.496 |
| *PDZD11* | 0.0000818 | 0.494 |
| *CGRRF1* | 0.000731 | 0.493 |
| *THAP12* | 0.0012254 | 0.492 |
| *TRIAP1* | 0.0015846 | 0.492 |
| *LOC102724112///PYM1* | 0.0009887 | 0.488 |
| *BLVRA* | 0.0010888 | 0.488 |
| *GAB1* | 0.0015288 | 0.485 |
| *BOLA3* | 0.0004125 | 0.482 |
| *PIK3C2A* | 0.0013111 | 0.482 |
| *YPEL5* | 0.0006559 | 0.48 |
| *BORCS7* | 0.0005184 | 0.479 |
| *COQ2* | 0.0003689 | 0.472 |
| *GTF2A1* | 0.0008115 | 0.469 |
| *RANBP2* | 0.001186 | 0.467 |
| *ZC3H13* | 0.0009325 | 0.462 |
| *SCAMP1* | 0.0008069 | 0.46 |
| *PIK3C2A* | 0.0015358 | 0.46 |
| *RAP1B* | 0.0015201 | 0.459 |
| *TMEM167B* | 0.0004489 | 0.453 |
| *SMIM19* | 0.0014232 | 0.453 |
| *MED13* | 0.0000334 | 0.45 |
| *C15orf61* | 0.0009232 | 0.447 |
| *MALSU1* | 0.0008922 | 0.446 |
| *IMPA1* | 0.0008949 | 0.445 |
| *ZNRD1* | 0.0006352 | 0.44 |
| *ABCE1* | 0.000932 | 0.435 |
| *FANCF* | 0.0007966 | 0.434 |
| *DYNLL1* | 0.0014074 | 0.422 |
| *TIMM8B* | 0.0011371 | 0.418 |
| *DUSP28* | 0.0015193 | 0.412 |
| *MFNG* | 0.0006121 | 0.411 |
| *TCEB1* | 0.000732 | 0.396 |
| *GOLGA7* | 0.001027 | 0.394 |
| *C11orf94* | 0.0012515 | 0.38 |
| *SRSF10* | 0.0001306 | 0.376 |
| *HYLS1* | 0.0013742 | 0.369 |
| *C20orf196* | 0.0011893 | 0.364 |
| *SERP1* | 0.0010801 | 0.353 |
| *HSBP1* | 0.0014428 | 0.353 |
| *ANKRD50* | 0.0008245 | 0.352 |
| *CASC4* | 0.001467 | 0.334 |
| *LEPROTL1* | 0.000696 | 0.328 |
| *HTRA2* | 0.0011899 | 0.325 |
| *LINC00324* | 0.0008317 | 0.28 |
| *LOC102723694* | 0.0004731 | -0.298 |
| *LOC102725526* | 0.0009893 | -0.335 |
| *ELMO2* | 0.0013135 | -0.343 |
| *MROH1* | 0.0012573 | -0.353 |
| *MIR6751///SYVN1* | 0.0014189 | -0.359 |
| *LINC01588* | 0.000912 | -0.37 |
| *ZCCHC7* | 0.0010522 | -0.397 |
| *ADD1* | 0.0001551 | -0.398 |
| *SPTAN1* | 0.0011216 | -0.398 |
| *NCOA3* | 0.0014652 | -0.423 |
| *WASF2* | 0.0004864 | -0.44 |
| *PARP14* | 0.0011689 | -0.457 |
| *CBX5* | 0.0013462 | -0.457 |
| *EZR* | 0.0003293 | -0.459 |
| *KAT6A* | 0.0008162 | -0.461 |
| *MYH9* | 0.0006795 | -0.482 |
| *HEXA* | 0.0007586 | -0.484 |
| *CD22* | 0.0011369 | -0.49 |
| *LOC102724250* | 0.0006736 | -0.495 |
| *KMT2A* | 0.0013703 | -0.495 |
| *GLG1* | 0.0005965 | -0.504 |
| *BRD4* | 0.0001212 | -0.517 |
| *AAK1* | 0.0013832 | -0.519 |
| *HIVEP2* | 0.000208 | -0.52 |
| *MYCBP2* | 0.0002174 | -0.53 |
| *ABLIM1* | 0.0006954 | -0.537 |
| *NCOA3* | 0.0003661 | -0.538 |
| *DDX21* | 0.0010808 | -0.539 |
| *GLG1* | 0.0006115 | -0.544 |
| *GHRLOS* | 0.001249 | -0.545 |
| *POM121C* | 0.0015858 | -0.547 |
| *CAMK1D* | 0.0008008 | -0.553 |
| *CHD2* | 0.0013232 | -0.562 |
| *BRD4* | 0.0010227 | -0.569 |
| *HERC1* | 0.0011012 | -0.577 |
| *TLN1* | 0.0007136 | -0.579 |
| *MYCBP2* | 0.0004002 | -0.583 |
| *IRAK3* | 0.0006407 | -0.604 |
| *LARP1* | 0.0007424 | -0.624 |
| *GTF2H2B* | 0.0000766 | -0.64 |
| *BRD4* | 0.00064 | -0.668 |
| *WNK1* | 0.0011906 | -0.731 |
| *DAPP1* | 0.0005657 | -0.745 |
| *LOC100128751* | 0.0006584 | -0.745 |
| *MALAT1* | 0.0009513 | -0.779 |
| *SIK3* | 0.0009602 | -0.824 |
| *EPG5* | 0.0008296 | -0.839 |
| *CEP350* | 0.001578 | -0.872 |
| *RBM6* | 0.0013538 | -0.992 |
