# Supplementary figures and images for: Analysis of Differentially Expressed Genes and Molecular Pathways in Familial Hypercholesterolemia Involved in Atherosclerosis: A Systematic and Bioinformatics Approach
Source: Front Genet. 2020 Jul 15;11:734. doi: 10.3389/fgene.2020.00734 (PMC7373787; doi:10.3389/fgene.2020.00734)

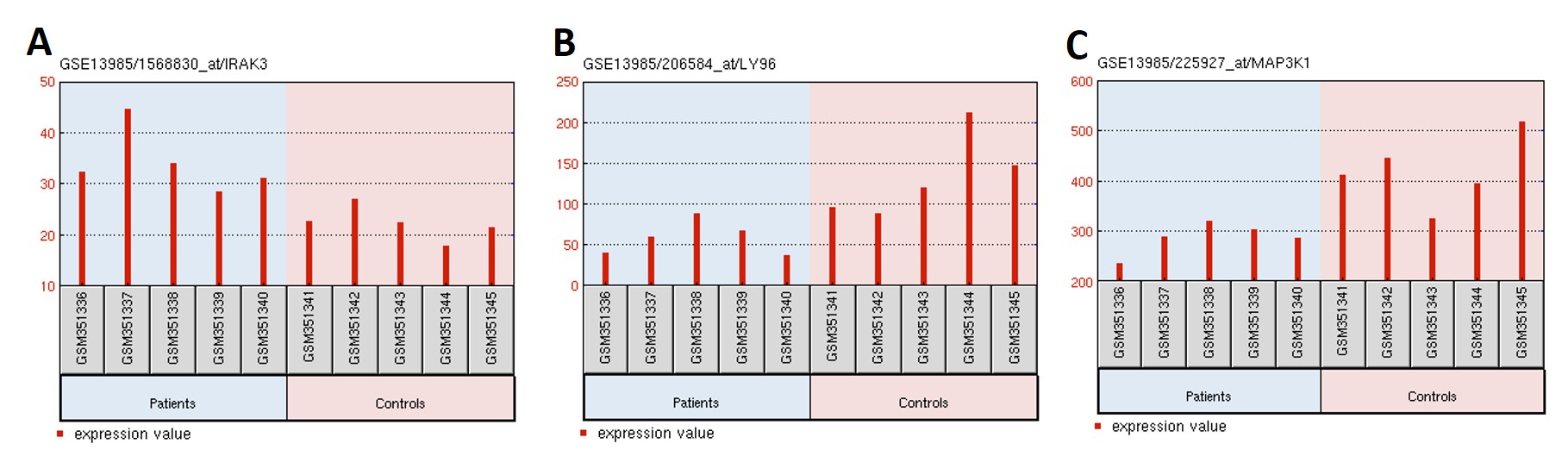

Supplement: Supplementary file 2 [file Image_1.JPEG]

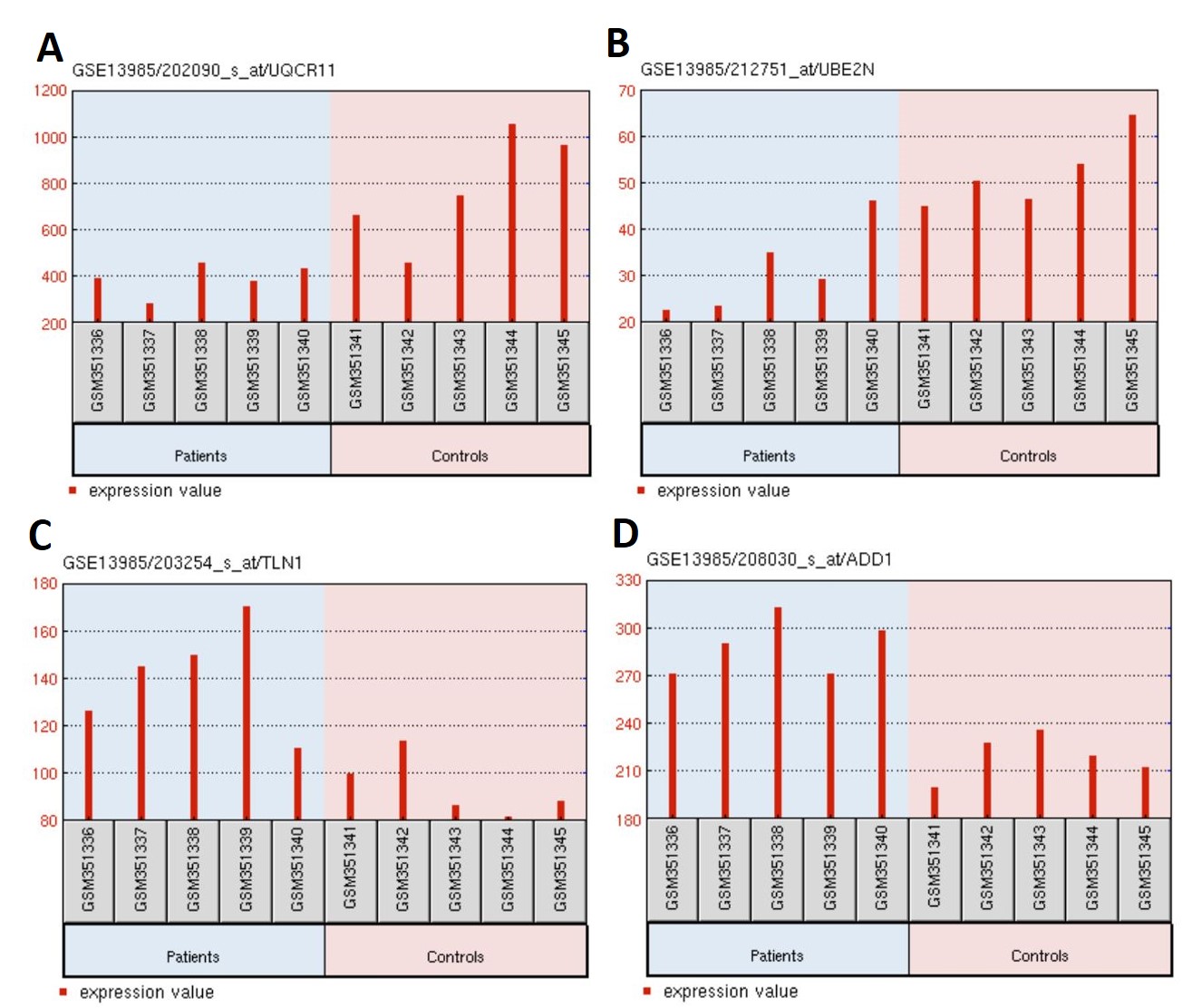

Supplement: Supplementary file 3 [file Image_2.JPEG]
